# Diagnosis and Assessment of Disease Activity in Takayasu Arteritis: A Childhood Case Illustrating the Challenge

**DOI:** 10.1155/2014/603171

**Published:** 2014-01-05

**Authors:** L. Watson, P. Brogan, I. Peart, C. Landes, N. Barnes, G. Cleary

**Affiliations:** ^1^Department of Paediatric Nephrology, Institute of Child Health, Alder Hey Children's NHS Foundation Trust Hospital, Eaton Road, Liverpool L12 2AP, UK; ^2^Department of Paediatric Rheumatology, Great Ormond Street NHS Hospital, London WC1N 3JH, UK; ^3^Department of Paediatric Cardiology, Alder Hey Children's NHS Foundation Trust Hospital, Eaton Road, Liverpool L12 2AP, UK; ^4^Department of Paediatric Radiology, Alder Hey Children's NHS Foundation Trust Hospital, Eaton Road, Liverpool L12 2AP, UK; ^5^Department of Paediatric Rheumatology, Alder Hey Children's NHS Foundation Trust Hospital, Eaton Road, Liverpool L12 2AP, UK

## Abstract

Takayasu Arteritis (TA) is a rare, debilitating large vessel vasculitis occurring in patients of all ages, including infants, but the disease most commonly presents in the third decade. Diagnosis is often delayed and consequently TA is associated with significant morbidity and mortality. Accurate methods of monitoring disease activity or damage are lacking and currently rely on a combination of clinical features, blood inflammatory markers, and imaging modalities. In this report we describe a case of a 14-year-old boy with childhood-onset TA who, despite extensive negative investigations, did indeed have on-going active large vessel vasculitis with fatal outcome. Postmortem analysis demonstrated more extensive and active disease than originally identified. This report illustrates and discusses the limitations of current modalities for the detection and monitoring of disease activity and damage in large vessel vasculitis. Clinicians must be aware of these limitations and challenges if we are to strive for better outcomes in TA.

## 1. Introduction

Takayasu Arteritis (TA) is a large vessel vasculitis of unknown aetiology that affects the aorta and its branches. The cause is unknown, but genetic contribution to disease susceptibility is increasingly recognised [1], whilst suggested links with tuberculosis infection remain unproven [2]. Diagnosis is often considerably delayed: in a recent paediatric classification criteria exercise, the mean time from symptom onset to diagnosis was 1.3 years (SD ± 1.6 years) [3]. Even when the diagnosis is secured, challenges occur in distinguishing between the acute or “active” phase of the illness and the chronic, stenotic phase when symptoms or signs are the result of tissue ischaemia from progressive arterial narrowing [4]. It is important to identify active disease since this requires immunosuppressive treatment, but it is unclear whether immunosuppression is effective in the late stage of the disease when a risk-benefit balance must be made [5]. Revascularisation procedures, such as angioplasty and/or stenting, or more invasive surgical interventions such as aortic bypass grafting are commonly required to provide relief from symptomatic ischaemia but are associated with significant life threatening risks and morbidity and are not completely effective in all cases [6, 7]. Moreover the risk of restenosis is high, particularly if the disease is active at the time of surgery [8–11]. We present a case that highlights the difficulties clinicians face in relation to the diagnosis and monitoring of large vessel vasculitis.

## 2. Case Presentation

Absence of retinal vessel pulsation was noted in a twelve-year-old boy during a routine ophthalmology assessment. He had presented with bilateral congenital glaucoma as an infant requiring surgical correction and hence long-term ophthalmology screening. At the age of eighteen months he had a right seventh nerve palsy that lasted 4 months and subsequently resolved; at the age of two years he developed acute, unexplained cardiac failure with evidence of dilated cardiomyopathy. He was commenced on digoxin, captopril, and frusemide and his cardiac contractility gradually recovered. The underlying cause of cardiomyopathy was never determined.

Between the ages of four and ten years he attended school full time. He had reduced stamina and a long history of calf claudication with a claudication distance of 200 m. He reported recurrent episodes of chest and left arm pain but no unexplained fevers and no cutaneous lesions. His growth parameters were normal (weight and height 50th centile for his age and sex). On examination, he had absent femoral, popliteal, dorsalis pedis, posterior tibialis, and brachial and radial pulses bilaterally. Centrally located abdominal and renal flank bruits were noted. Routine laboratory markers of inflammation (white cell count, erythrocyte sedimentation rate, C reactive protein) were normal, as were routine haematological and biochemistry panels. Antinuclear and antineutrophil cytoplasmic antibodies were negative. Peripheral blood cytokine analysis (TNF alpha, IL-6, and IL10) was normal; in addition he had a normal circulating endothelial cell (CEC) count of 20 cells per mL of whole blood [12]. It was noted however that he had a reversed CD4 : CD8 ratio (32% : 40%, resp.). 

**Figure 1:**
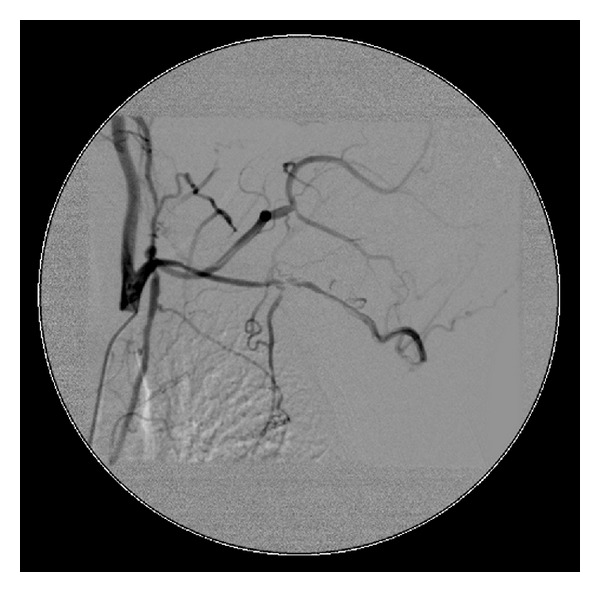
Angiography demonstrating severe stenosis of the aortic branches and complete occlusion below the level of the mesenteric artery.

**Figure 2:**
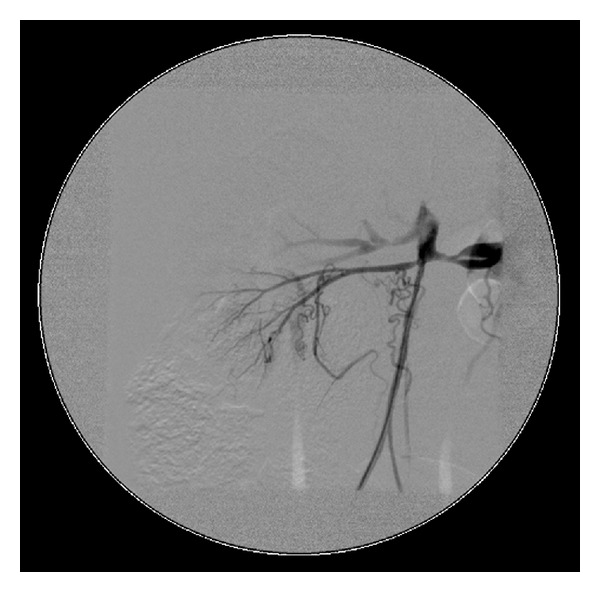
Angiography demonstrating the thread-like aorta up to the level of the common iliac arteries.

**Figure 3:**
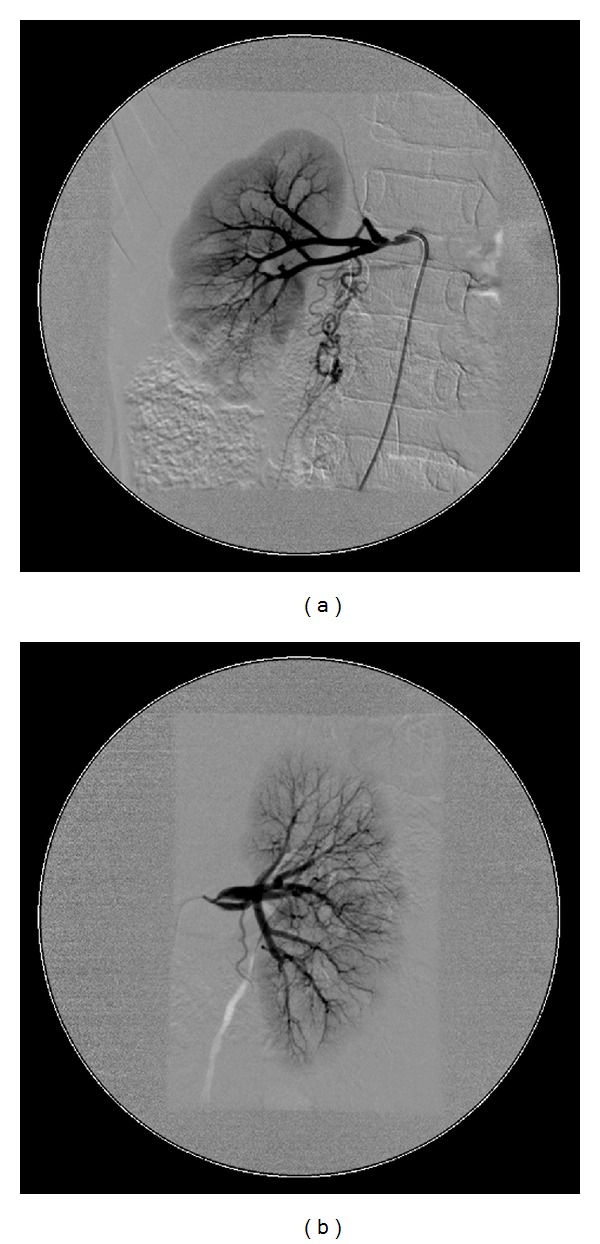
Angiography demonstrating severe renal artery stenosis and multiple intrarenal vessel narrowing and collateral blood vessels branching away from the renal artery.

**Figure 4:**
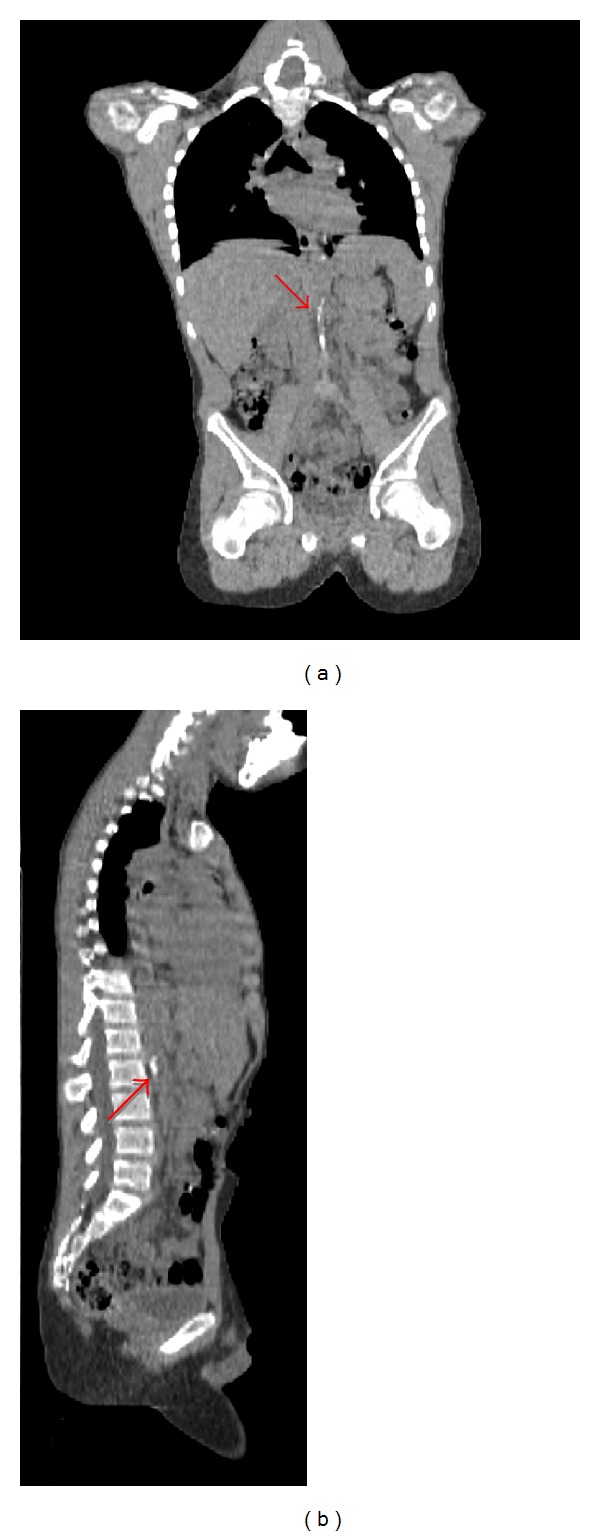
An unenhanced computerized tomography (CT) image (coronal and sagittal reformatted) showing aortic calcification (arrow).

Multiple radiological investigations were undertaken to assess for on-going active large vessel vasculitis and/or late stenotic sequelae. Radiological investigations collectively revealed widespread arterial disease with occlusion of the subclavian, common iliac, and proximal tibial and peroneal arteries (Figures 1, 2, and 3). On computer tomography (CT) angiography, and conventional catheter digital subtraction arteriography the descending abdominal aorta was irregular and tapered distal to the renal arteries with severe proximal bilateral renal artery stenosis (Figure 4). The abdominal aorta was thread-like up to the level of the common iliac arteries with some irregular calcific plaque demonstrated on the CT scan (Figures 2 and 4). He had an extensive collateral circulation throughout his arterial tree supplying his limbs. 

A half body PET-CT (positron emitting tomography, PET) was performed using fludeoxyglucose (FDG) that showed no uptake of FDG in the large vessels. Echocardiogram showed slight left ventricular impairment with a normal aortic arch, confirmed on angiography, and the coronary arteries appeared normal although selective coronary arteriography was not undertaken.

The clinical history, symptoms, absence of acute phase response, and imaging appearances were compatible with a large vessel vasculitis, currently inactive and in the late stenotic phase of the disease. The exact time of disease onset was unknown. The only positive investigation was a reversed T cell (CD4 : CD8) ratio, and with no FDG uptake on PET-CT, the presence of extensive arterial stenotic lesions, absence of acute phase response including normal peripheral blood cytokines, normal CECs, and aortic intramural calcification, we considered the disease to be in the late fibrotic/stenotic stage. It was therefore felt that immunosuppression was unlikely to confer any benefit. Since there was no obvious angioplasty/revascularisation target; management consisted of a careful and graded return to activity and lifestyle advice to minimise conventional cardiovascular risk factors in the future.

At the age of 14 years he collapsed suddenly; resuscitation attempts were unsuccessful. Postmortem examination revealed severe left ventricular hypertrophy and 70% occlusion of the coronary arteries. The abdominal aorta was narrowed with a patchy and sparse chronic inflammatory cell infiltrate and evidence of active inflammation within the vasa vasorum of the distal aorta. There was marked intimal fibrosis with calcification and collateral vessels, as noted on previous imaging.

## 3. Discussion

Diagnosing TA relies on clinical presentation, characteristic structural arterial abnormalities, and evidence of inflammatory vasculopathy on imaging or histology. A classification of childhood TA exists (Table 1); however the major clinical challenge when considering the disease is distinguishing this inflammatory large vessel vasculitis from other noninflammatory vasculopathies, since the differential diagnosis is broad (Table 2). This issue is particularly difficult when the disease is in the late stenotic phase, where acute vasculitic disease activity may be minimal or absent.

Since clinical features may be very nonspecific or even absent, delay in the diagnosis of TA is unfortunately very common, sometimes of several years [13]. In this case the onset of inflammatory arteritis may have been present for some time, as collateral vessels take several years to develop, and our patient had an extensive collateral vascular system.

Once the diagnosis of TA is secured, there is currently no gold standard investigation that can be used for monitoring disease activity. A previous study involving 10 Indian patients with TA demonstrated that postmortem analysis identified more active disease than what was detectable clinically [14]. Meticulous monitoring of this condition is therefore essential but poses significant challenges. Clinicians currently combine clinical features with acute phase reactants such as the erythrocyte sedimentation rate and/or C reactive protein; imaging techniques such as PET-CT [15, 16], contrast enhanced MR [17], and CT angiography [18, 19]; and formal digital subtraction arteriography, perceived by most to be the gold standard for defining lumenography to monitor for disease activity and for stenotic sequelae. 

Routinely used acute phase reactants may be elevated in the early or active phases of the disease; however their sensitivity and specificity remain poor. In a study by Dagna et al., receiver operating characteristic curves showed an area under the curve value of 0.75 and 0.68 for ESR and CRP, respectively, demonstrating that they were inferior to a novel biomarker, pentraxin-3 [20, 21]. Pentraxin-3 is a protein rapidly produced by many cells, particularly endothelial cells, in response to inflammatory signals [22] and is believed to be an early marker of inflammatory activity in the vessels. Reversal of T cell CD4 : CD8 balance with an increase in the number of CD8 cells has been demonstrated to be a useful marker of disease activity in this condition, as seen in our patient [23]. Whilst emerging data support the use of circulating endothelial cells in small and medium vessel vasculitides [24, 25], their role as potential biomarkers in large vessel vasculitides remains unknown. Large studies assessing the role of anti-CEC antibodies in TA are also lacking [24].

The uptake of 18F-fluorodeoxyglucose on PET imaging is reported as a potentially highly sensitive and accurate method for assessing disease activity in patients with TA. In studies of adult patients with TA (the largest study involving 39 participants) the sensitivity and specificity of 18FDG-PET scanning was shown to be 73–100% and 83–92%, respectively [26–28]. Contrast-enhanced MRI with late gadolinium administration may detect increased arterial wall thickness with evidence of late gadolinium enhancement, suggestive of active large vessel vasculitis [17, 29]. CT angiography is able to detect vessel wall alterations and define luminal changes and may be useful during diagnosis and subsequent monitoring, although this lacks an ability to define active vasculitis [17, 29–31]. In our case, absence of FDG uptake on PET-CT leads us to conclude that contrast induced MRA was unlikely to add further information, and hence this test was not undertaken.

Digital subtraction catheter arteriography is regarded by most as a gold standard for defining lumenography, of particular importance in the assessment of patients potentially requiring revascularisation procedures [32]. Surgical techniques such as angioplasty and bypass are reserved for symptomatic use to restore circulation but do not alter the underlying disease process and are associated with a higher morbidity and even mortality if performed during active disease [33, 34]. This latter point emphasises the importance of accurately defining disease activity if considering a revascularisation process.

In summary, TA can present in childhood and the diagnosis is often significantly delayed despite advances in laboratory biomarkers and imaging to detect vascular inflammation and anatomy. We present a paediatric case with fatal outcome where postmortem studies revealed the true extent of on-going vasculitic disease activity, and extent of arterial injury, particularly in relation to coronary arterial involvement. We suggest that one of the main reasons the prognosis for TA in the paediatric population remains (arguably) unacceptably poor is through diagnostic delay. If we are to influence this, clinicians must consider the diagnosis earlier and be aware of the limitations of the current technologies for the detection of active large vessel vasculitis. A concerted, international effort of clinical data collection in disease registries and challenging treatments using clinical trials is needed to improve the evidence base for the diagnosis and management of large vessel vasculitis in young patients.

## Figures and Tables

**Table 1:** EULAR/PRINTO/PRES criteria and classification definition of Takayasu Arteritis.

Mandatory criteria	
Angiographic abnormality	Angiography (conventional, CT, and MRI) of the aorta, its main branches or pulmonary arteries showing aneurysm/dilatation, narrowing, occlusion, or thickened arterial wall, not due to any other causes

Additional criteria (need one of the five)	

(1) Pulse deficit or claudication	Lost/decreased/unequal peripheral artery pulse
	Symptoms of claudication: focal muscle pain induced by physical activity
(2) Blood pressure discrepancy	Discrepancy of four-limb systolic blood pressure >10 mmhg in any limb
(3) Bruits	Audible murmurs or palpable thrills over large arteries
(4) Hypertension	Systolic/diastolic blood pressure >95th centile for height
(5) Acute phase reactant	Erythrocyte sedimentation rate (ESR) >20 mm per hour or C reactive protein (CRP) above normal

**Table 2:** Differential diagnosis of Takayasu Arteritis.

Classification	Subtype
Primary inflammatory vasculitides	Giant cell arteritis
	Kawasaki disease
	Polyarteritis nodosa
	Wegener's granulomatosis disease

Secondary inflammatory vasculitides	Rheumatic fever
	Sarcoidosis
	SLE
	Behcet's disease
	Spondyloarthropathies

Noninflammatory vascular disease	Fibromuscular dysplasia
	Congenital aortic abnormalities
	Inherited connective tissue disorders
	Neurofibromatosis

Other	Sepsis
	Malignancy
	Human immunodeficiency virus
	Syphilis
	Tuberculosis
	Radiation fibrosis

## References

[1] Morishita KA, Rosendahl K, Brogan PA (2011). Familial Takayasu arteritis—a pediatric case and a review of the literature. *Pediatric Rheumatology*.

[2] Arnaud L, Cambau E, Brocheriou I (2009). Absence of Mycobacterium tuberculosis in arterial lesions from patients with Takayasu’s arteritis. *Journal of Rheumatology*.

[3] Ruperto N, Ozen S, Pistorio A (2010). EULAR/PRINTO/PRES criteria for Henoch-Schönlein purpura, childhood polyarteritis nodosa, childhood Wegener granulomatosis and childhood Takayasu arteritis: Ankara 2008. Part I: overall methodology and clinical characterisation. *Annals of the Rheumatic Diseases*.

[4] Maksimowicz-McKinnon K, Clark TM, Hoffman GS (2007). Limitations of therapy and a guarded prognosis in an American cohort of Takayasu arteritis patients. *Arthritis and Rheumatism*.

[5] Mukhtyar C, Guillevin L, Cid MC (2009). EULAR recommendations for the management of large vessel vasculitis. *Annals of the Rheumatic Diseases*.

[6] Rao SA, Mandalam KR, Rao VR (1993). Takayasu arteritis: initial and long-term follow-up in 16 patients after percutaneous transluminal angioplasty of the descending thoracic and abdominal aorta. *Radiology*.

[7] Kim HJ, Lee C-S, Kim JS (2011). Outcomes after endovascular treatment of symptomatic patients with Takayasu’s arteritis. *Interventional Neuroradiology*.

[8] Kerr GS, Hallahan CW, Giordano J (1994). Takayasu arteritis. *Annals of Internal Medicine*.

[9] Son JW, Koh KK, Dang Q, Choi IS, Shin EK (1998). Recurrent restenosis following stent and rotational atherectomy of coronary artery stenosis in Takayasu’s arteritis. *International Journal of Cardiology*.

[10] Amano J, Suzuki A (1991). Coronary artery involvement in Takayasu’s arteritis: collective review and guideline for surgical treatment. *Journal of Thoracic and Cardiovascular Surgery*.

[11] Pajari R, Hekali P, Harjola P-T (1986). Treatment of Takayasu’s arteritis: an analysis of 29 operated patients. *Thoracic and Cardiovascular Surgeon*.

[12] Clarke LA, Hong Y, Eleftheriou D (2010). Endothelial injury and repair in systemic vasculitis of the young. *Arthritis and Rheumatism*.

[13] Watts R, Al-Taiar A, Mooney J, Scott D, MacGregor A (2009). The epidemiology of Takayasu arteritis in the UK. *Rheumatology*.

[14] Sharma BK, Jain S, Radotra BD (1998). An autopsy study of Takayasu arteritis in India. *International Journal of Cardiology*.

[15] Sager S, Yilmaz S, Ozhan M (2012). F-18 Fdg PET/CT findings of a patient with Takayasu arteritis before and after therapy. *Molecular Imaging and Radionuclide Therapy*.

[16] Cheng Y, Lv N, Wang Z, Chen B, Dang A (2013). 18-FDG-PET in assessing disease activity in Takayasu arteritis: a meta-analysis. *Clinical and Experimental Rheumatology*.

[17] Papa M, De Cobelli F, Baldissera E (2012). Takayasu arteritis: intravascular contrast medium for MR angiography in the evaluation of disease activity. *American Journal of Roentgenology*.

[18] Schmidt WA (2013). Imaging in vasculitis. *Best Practice & Research Clinical Rheumatology*.

[19] Zhu FP, Luo S, Wang ZJ, Jin ZY, Zhang LJ, Lu GM (2012). Takayasu arteritis: imaging spectrum at multidetector CT angiography. *British Journal of Radiology*.

[20] Moriwaki R, Numano F (1992). Takayasu arteritis: follow-up studies for 20 years. *Heart and Vessels*.

[21] Dagna L, Salvo F, Tiraboschi M (2011). Pentraxin-3 as a marker of disease activity in takayasu arteritis. *Annals of Internal Medicine*.

[22] Cieślik P, Hrycek A (2012). Long pentraxin 3 (PTX3) in the light of its structure, mechanism of action and clinical implications. *Autoimmunity*.

[23] Uppal SS, Verma S (2003). Analysis of the clinical profile, autoimmune phenomena and T cell subsets (CD4 and CD8) in Takayasu’s arteritis: a hospital-based study. *Clinical and Experimental Rheumatology*.

[24] Eichhorn J, Sima D, Thiele B (1996). Anti-endothelial cell antibodies in Takayasu arteritis. *Circulation*.

[25] Wang H, Ma J, Wu Q, Luo X, Chen Z, Kou L (2011). Circulating B lymphocytes producing autoantibodies to endothelial cells play a role in the pathogenesis of Takayasu arteritis. *Journal of Vascular Surgery*.

[26] Karapolat I, Kalfa M, Keser G (2012). Comparison of F18 FDG PET/CT findings with current clinical disease status in patients with Takayasu's arteritis. *Clinical and Experimental Rheumatology*.

[27] Tezuka D, Haraguchi G, Ishihara T (2012). Role of FDG PET-CT in Takayasu arteritis: sensitive detection of recurrences. *JACC: Cardiovascular Imaging*.

[28] Fuchs M, Briel M, Daikeler T (2012). The impact of18F-FDG PET on the management of patients with suspected large vessel vasculitis. *European Journal of Nuclear Medicine and Molecular Imaging*.

[29] Schneeweis C, Schnackenburg B, Stuber M (2012). Delayed contrast-enhanced MRI of the coronary artery wall in takayasu arteritis. *PLoS One*.

[30] Eshet Y, Pauzner R, Goitein O (2011). The limited role of MRI in long-term follow-up of patients with Takayasu’s arteritis. *Autoimmunity Reviews*.

[31] Yadav MK (2007). Takayasu arteritis: clinical and CT—angiography profile of 25 patients and a brief review of literature. *Indian Heart Journal*.

[32] Khandelwal N, Kalra N, Garg MK (2011). Multidetector CT angiography in Takayasu arteritis. *European Journal of Radiology*.

[33] Hu J, Huang H, Zhang X (2012). Stent placement for treatment of long segment (>/=80mm) carotid artery stenosis in patients with takayasu disease. *Journal of Vascular and Interventional Radiology*.

[34] Saadoun D, Lambert M, Mirault T (2012). Retrospective analysis of surgery versus endovascular intervention in Takayasu arteritis a multicenter experience. *Circulation*.

